# Genetics of idiopathic inflammatory myopathies: insights into disease pathogenesis

**DOI:** 10.1097/BOR.0000000000000652

**Published:** 2019-09-19

**Authors:** Simon Rothwell, Hector Chinoy, Janine A. Lamb

**Affiliations:** a Centre for Musculoskeletal Research, Faculty of Biology, Medicine and Health; b National Institute for Health Research Manchester Biomedical Research Centre, Manchester University NHS Foundation Trust, University of Manchester, Manchester; c Rheumatology Department, Salford Royal NHS Foundation Trust, Manchester Academic Health Science Centre, Salford; d Division of Population Health, Health Services Research & Primary Care, Faculty of Biology, Medicine and Health, University of Manchester, Manchester, UK

**Keywords:** genetics, genome-wide association study, human leukocyte antigen, idiopathic inflammatory myopathies, myositis

## Abstract

To review the advances that have been made in our understanding of the genetics of idiopathic inflammatory myopathies (IIM) in the past 2 years, with a particular focus on dermatomyositis and polymyositis.

**Recent findings:**

Fine-mapping studies in the major histocompatibility complex region in Caucasian and Korean populations have identified novel human leukocyte antigen (HLA) variants that are associated with autoantibody subgroups in IIM. Differences in HLA associations have been identified between Caucasian adult-onset and juvenile-onset patients with anti-TIF1 autoantibodies, suggesting distinct aetiologies in these patients. For some autoantibodies, the strongest associations identified are specific amino acid positions within HLA molecules, providing mechanistic insights into disease pathogenesis.

A meta-analysis combining data from four seropositive rheumatic diseases identified 22 novel non-HLA associations in IIM, of which seven were previously reported at suggestive significance in IIM. A genome-wide association study conducted in the Japanese population identified a significant association with *WDFY4* in patients with clinically amyopathic dermatomyositis.

**Summary:**

Considerable progress has been made in understanding the genetics of IIM, including differences in clinical and autoantibody subgroups. As research continues, there should be a focus to increase statistical strength and precision by conducting meta-analyses and trans-ethnic studies.

## INTRODUCTION

The idiopathic inflammatory myopathies (IIM), collectively known as myositis, are a group of rare autoimmune diseases. They are characterized primarily by skeletal muscle weakness and muscle inflammation, and commonly present with extramuscular manifestations such as skin rash, interstitial lung disease, polyarthritis and cancer. IIM are heterogeneous, and are clinically subclassified as dermatomyositis, inclusion body myositis (IBM), immune-mediated necrotizing myopathy (IMNM), polymyositis and anti-synthetase syndrome (ASS) [1,2]. IIM are complex diseases thought to be initiated by immune activation following specific environmental events in genetically predisposed individuals. Significant progress has been recently made in studying the genetics of IIM, and we are beginning to understand more about the genetic architecture of these rare diseases. The present article reviews the advances that have been made in the past 2 years in our understanding of the genetics of IIM, in particular dermatomyositis and polymyositis. 

**Box 1:**
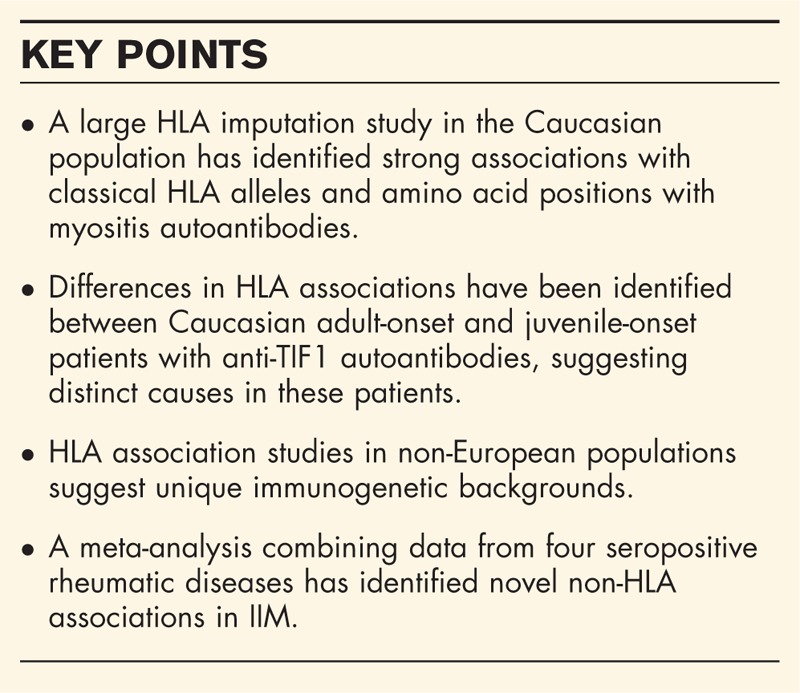
no caption available

## MAJOR HISTOCOMPATIBILITY COMPLEX GENETICS

In IIM, as in many other autoimmune and immune-mediated diseases, the strongest genetic risk for disease susceptibility lies within the major histocompatibility complex (MHC), and is thought to localize to specific human leukocyte antigen (*HLA*) alleles [3]. The 8.1 ancestral haplotype (AH), a common haplotype of extensive linkage disequilibrium in Caucasian populations, is a strong risk factor for IIM and many other autoimmune diseases. Identifying which genes in the MHC region are causal is an ongoing subject of debate.

Distinct *HLA* associations have been described in IIM in different populations, clinical subgroups and with specific clinical features, however the strongest *HLA* associations are found when stratifying by autoantibody status [3]. Using data from a large recent genetic association study in IIM, we correlated autoantibody type with imputed *HLA* genotype in patients to identify novel risk variants in the MHC region that confer susceptibility to IIM autoantibodies [4^▪▪^]. SNP2HLA was used to impute HLA gene and amino acid variants using a large imputation reference panel [5]. Associations with alleles of the 8.1 AH were observed for patients with anti-Jo-1, anti-PM/Scl and anti-cN1A autoantibodies. Anti-HMGCR and anti-Mi-2 were the only autoantibodies tested that were associated with *HLA* alleles not forming part of the 8.1 AH. For patients with anti-Jo-1 autoantibodies, there was an additional independent effect of the class I allele *HLA-B*^*∗*^*08 : 01*. This allele also forms part of the 8.1 AH suggesting that multiple independent risk factors within this haplotype confer risk of autoimmunity. Indeed, other genes on the 8.1 AH may predispose individuals to immune-mediated diseases, such as NF-κB and tumour necrosis factor-α polymorphisms [6,7], and copy number variants of complement genes [8]. In addition, a recent study in anticitrullinated protein antibody (ACPA) positive rheumatoid arthritis (RA) has identified a risk within *HLA-DOA*, a nonclassical *HLA* gene, suggesting *HLA* alleles not imputed by SNP2HLA may contribute to disease risk [9].

There is also evidence of genetic differences between adults and juveniles with the same autoantibody, as has been reported previously with anti-HMGCR autoantibodies [9,10]. Of particular interest in IIM are the different haplotype associations for adult-onset and juvenile-onset patients with anti-TIF1 autoantibodies [4^▪▪^], where there is a strong association with cancer in adult-onset disease but not juvenile-onset disease [11,12]. After stratifying by age, an association between anti-TIF1 autoantibodies and the 8.1 AH was present in juvenile-onset patients but not adult-onset patients. This suggests that there may be distinct causes that differentiate adult-onset and juvenile-onset disease. For example, genetic modifications including somatic mutations and loss of heterozygosity in the *TRIM33* gene encoding TIF1γ have recently been reported in tumours in adults with cancer-associated myositis [13^▪^]. In juvenile-onset patients there may be environmental triggers such as infection [14,15].

To refine the associations within the *HLA* region, SNP2HLA imputes amino acid locations within HLA proteins, which may give mechanistic insights in to disease pathogenesis. For some IIM autoantibodies, amino acid locations were more strongly associated than classical alleles alone [4^▪▪^]. For example, amino acid position 74 of HLA-DRB1, which faces inwards of the peptide binding groove in HLA DR molecules, was the most strongly associated amino acid position in patients with anti-Jo-1, anti-PM/Scl and anti-cN1A autoantibodies. This location has been implicated in other autoimmune diseases in Caucasian individuals [16–18]. Identification of shared amino acid signatures across different ethnicities may give insight in to whether these positions are functionally important for susceptibility to certain autoantibody profiles [19].

Many of these risk associations have been validated in a large phenome-wide association study (PheWAS), which examined relationships between classical *HLA* variants and amino acid positions, and a range of human disease phenotypes taken from electronic health records in European ancestry individuals [20]. An online catalogue reported associations of *HLA-DRB1*^*∗*^*03 : 01* and *HLA-B*^*∗*^*08 : 01* in a range of autoimmune diseases, including strong associations with polymyositis and dermatomyositis.

Another study investigating *HLA*–autoantibody associations has been conducted in the Korean population. *HLA-DRB1* and *HLA-DPB1* alleles were typed in 179 IIM patients with dermatomyositis (*n* = 129) or polymyositis (*n* = 50) [21^▪^]. Correlations between individual *HLA* alleles and anti-MDA5, anti-aminoacyl-tRNA synthetase (anti-ARS), anti-TIF1, anti-SRP and anti-Mi-2 autoantibodies were investigated. Strikingly, the observed associations suggest a unique immunogenetic background of Korean patients with myositis compared to the Caucasian population. Differences can also be seen in the frequency of autoantibodies. The most common autoantibody in this study was anti-MDA5 in 26.8% of patients tested (*n* = 48), and a strong association was reported with *HLA-DRB1*^*∗*^*12 : 02* (*P*_corr_ = 0.001; OR = 5.46; 95% CI, 2.67–11.20). In Rothwell *et al.*[4^▪▪^], anti-MDA5 autoantibodies were detected in 1.7% of patients tested (*n* = 35); however, no *HLA* allele was associated at a study wide significance level of *P* < 2.9 × 10^–5^. Notably, *HLA-DRB1*^*∗*^*12 : 02* is very rare in Caucasian populations (http://www.allelefrequencies.net) [22]. There was no strong association with *HLA-DRB1*^*∗*^*03 : 01* on the 8.1 AH in Korean IIM patients, likely because of the rarity of this haplotype in Asian populations [23,24]. An association was identified with anti-ARS autoantibodies and *HLA-DRB1*^*∗*^*08 : 03* (*P*_corr_ = 0.02; OR = 4.15; 95% CI, 1.89–9.09). The only antibody with the same association in the Korean population and Caucasians is anti-Mi-2 (*HLA-DRB1*^*∗*^*07 : 01*, *P*_corr_ = 0.0003; OR = 10.23; 95% CI, 3.81–27.51). Although classical *HLA* associations differ across ethnicities, there may be features of risk alleles or haplotypes that are shared across populations. *HLA* fine-mapping analysis results reflect the linkage disequilibrium structure of examined populations, therefore including different ethnic populations could contribute to identification of additional independent association signals by breaking down population specific haplotype blocks. Further work investigating ethnic heterogeneity within the MHC in IIM therefore may be informative.

*HLA* associations have been described in other Asian populations, including the Japanese [25] and Vietnamese [26]. There have also been multiple candidate gene studies in the Han Chinese population, reporting novel associations with *HLA-DRB1* and *HLA-DPB1* alleles and clinical features of disease [23,27], and with anti-MDA5 autoantibodies [27,28]. Because of the smaller studies in the Han Chinese population, genetic associations with clinical subgroup or autoantibody status are often conflicting, or underpowered. Conducting a meta-analysis or systematic review of these studies to identify genuine associations in this population could be revealing.

## GENOME-WIDE ASSOCIATIONS

The largest genetic association study to date in IIM was conducted using the Immunochip including 2566 patients with polymyositis, dermatomyositis, juvenile dermatomyositis (JDM) and IBM [29]. The *HLA* region and *PTPN22* gene were both associated at genome-wide significance, and there were a number of loci that reached the study-wide significance threshold of *P* < 2.25 × 10^−5^. A recent meta-analysis combined GWAS data from four systemic seropositive rheumatic diseases; systemic sclerosis, systemic lupus erythematosus, RA, and IIM [30^▪▪^]. The authors report 26 shared genome-wide significant loci, five of which have never been associated with these diseases before. IIM contributed to 22 of the observed associations, and interestingly seven of these associations were the same as those found in the Immunochip study reaching study-wide significance (*PTPN22, NAB1, STAT4, DGKQ, FAM167A-BLK, YDJC*). *TYK2* has also been reported before in IIM in a separate candidate gene study [31]. Validating what we already know about the genetic architecture of autoimmune disease, many of the associations identified are significantly enriched in regulatory regions in relevant immune cells, and many are expression quantitative trait loci (eQTL) for genes involved in the immune system [32]. This analysis strengthens our confidence in associations previously reported as suggestive in IIM. Many of the reported associations are enriched in drug targets either being tested or currently used in these diseases, showing how genetic data can be used to identify potential novel therapies in IIM or for drug repositioning.

Until recently, GWAS conducted in IIM have been in the Caucasian population [29,33,34]. The first GWAS in the Asian population was recently conducted in a Japanese cohort comprising 236 patients with polymyositis and 340 patients with dermatomyositis, of which 33 patients had clinically amyopathic dermatomyositis (CADM) [35^▪▪^]. Interestingly, there was no association with the MHC region in contrast to the strong genetic risk seen in Caucasian studies. No genome-wide significant associations (*P* < 5 × 10^−8^) were found with the total IIM cohort, or the dermatomyositis and polymyositis subgroups. A significant association with a SNP intronic of *WDFY4* was reported in 33 patients with CADM (rs7919656; *P* = 1.5 × 10^−8^; OR = 3.87; 95% CI, 2.23–6.55). This variant is associated with higher expression of a truncated *WDFY4* isoform and increased expression of NF-κB associated genes. Functional analysis in IIM suggests that WDFY4 interacts with pattern recognition receptors, with isoforms of *WDFY4* differentially augmenting NF-κB signalling. Notably, the lead association in the Japanese CADM subgroup was not significant in a separate analysis of 21 European CADM cases, however independent variants in *WDFY4* were seen at nominal significance (rs2889697, *P* = 0.0058).

## GENE–ENVIRONMENT ASSOCIATIONS

IIM are thought to be initiated by immune activation following specific environmental events in genetically predisposed individuals. As yet, there are few validated environmental risk factors identified for IIM. As extensive genetic data are being generated on more myositis patients and broader clinical data collected, we are able to replicate some of the gene–environment interactions that have previously been suggested in IIM.

Smoking is thought to be a risk factor in IIM for the development of anti-Jo-1 autoantibodies [36]. A recent study by Schiffenbauer *et al.*[37] explored this association, showing that smoking was associated with an increased risk of developing polymyositis, anti-Jo-1 and anti-synthetase autoantibodies, with the greatest risk attributable to those who smoked and carried the *HLA-DRB1*^*∗*^*03 : 01* allele. The risk of only one of these two risk-factors was intermediate. Interestingly, an inverse association was found with anti-TIF1, where smokers with *HLA-DRB1*^*∗*^*03 : 01* were less likely to have these autoantibodies.

A further environmental risk factor for IIM appears to be ultraviolet (UV) radiation, as suggested by data showing that the likelihood of developing dermatomyositis over polymyositis significantly increases towards the equator [38], attributable to higher UV exposure [39]. In particular, one study has shown that UV radiation is associated with the relative proportion of individuals with anti-Mi-2 autoantibodies, where patients’ characteristic skin changes are more prominent on the sun-exposed parts of the body [40]. A recent study investigated whether there may be genetic risk factors that could explain the latitudinal gradient of dermatomyositis prevalence, in addition to UV exposure [41]. The authors analysed the association of latitude with classical *HLA* alleles and SNPs associated with IIM and dermatomyositis autoantibodies in healthy control subjects. The authors confirm an increase in prevalence of dermatomyositis towards the equator, and report a novel finding that the frequency of anti-TIF1 autoantibodies is negatively correlated with latitude. In addition, *HLA* alleles significantly associated with anti-Mi-2 and anti-TIF1-γ autoantibodies also were strongly negatively associated with latitude, suggesting that genetic background, in addition to UV exposure, may contribute to the distribution of dermatomyositis.

## GENE EXPRESSION STUDIES IN IDIOPATHIC INFLAMMATORY MYOPATHIES

As the cost of RNA sequencing drops, we will begin to understand in finer detail genes that are differentially expressed in subgroups of IIM, and between different target tissues. A recent study investigated differential gene expression profiles in T cell subsets that differ between polymyositis and dermatomyositis [42]. Inflammatory cells infiltrating muscle fibres is a hallmark feature of IIM. In polymyositis, there is a predominance of CD8+ T cells, whereas in dermatomyositis CD4+ T cells predominate, along with plasmacytoid dendritic cells and B cells. RNA-sequencing was conducted on CD4+ T cells (polymyositis = 8 and dermatomyositis = 7) and CD8+ T cells (polymyositis = 4 and dermatomyositis = 5) isolated from peripheral blood mononuclear cells. Although overall gene expression was similar between polymyositis and dermatomyositis in T cells, differential gene expression analysis revealed 176 genes expressed in CD8+ T cells, that differ between patients with polymyositis compared to dermatomyositis. Many of these genes are involved in lymphocyte migration and T-cell differentiation. In contrast, in the CD4+ analysis, only two genes were significantly differentially expressed; *ANKRD55* and *S100B.* Interestingly, *ANKRD55* is a strong genetic risk factor for a number of autoimmune diseases [43–46].

A study by Pinal-Fernandez *et al.*[47] used RNA-sequencing to quantify the expression of IIM autoantigens in muscle and regenerating muscle, and to investigate whether autoantigen expression correlates with the corresponding autoantibody. RNA from 106 muscle biopsies from patients with IIM autoantibodies (anti-HMGCR, anti-Jo-1, anti-NXP2, anti-TIF1γ, anti-Mi-2, anti-SRP, and anti-MDA5) and 20 healthy controls were sequenced. All IIM autoantigens studied were expressed in muscle biopsies, and the levels positively correlated with markers of muscle regeneration. Autoantigens were also expressed in regenerating mouse muscles and in cultured human myoblasts. Notably, the expression of IIM autoantigens was not associated with the presence of the corresponding autoantibody. It is still unknown why one autoantigen is preferentially targeted by the immune system in patients with IIM.

Many studies in IIM report a strong interferon (IFN) signature in which there is upregulation of type I IFN inducible transcripts and activation of the type I IFN pathway [48]. This is in keeping with the function of genes that have been reported in the GWAS meta-analysis discussed above, such as *NAB1*, *TYK2*, *PTPN11*, *IRF5*, and *IRF8*[30^▪▪^]. Moreover, recent studies have stratified patients by clinical subgroup and found differences in IFN signature in muscle biopsies [49,50]. Dermatomyositis is commonly associated with a type I-IFN signature, but distinct associations with IBM and ASS and type-II IFN, and the lack of an IFN signature in IMNM, suggest differences in pathogenesis and potential for targeted therapies in IIM.

## CONCLUSION

**FIGURE 1:**
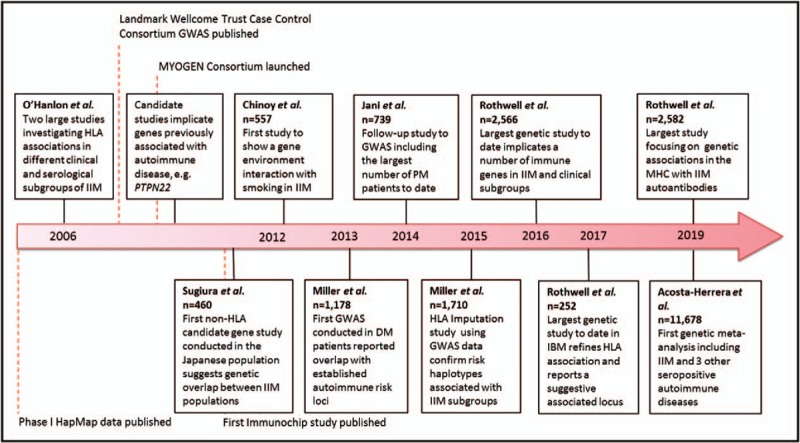
Timeline of key genetic studies published in IIM. Landmark studies that have been published in IIM genetics research, and the progress that has been made since the last IIM genetics review in 2016 [52].

Considerable progress has been made in our understanding of the genetics of IIM, as summarized in Fig. 1. The strongest genetic risk lies within the MHC region, and we are beginning to understand in greater detail how heterogeneity within this region contributes to disease susceptibility in different clinical and autoantibody subgroups of IIM. As research continues in different populations, there should be a focus to increase statistical strength and precision by combining the results in trans-ethnic meta-analyses. As we have seen from genome-wide association studies, leveraging power from other related diseases, either by meta-analysis or novel statistical techniques [51], will continue to reveal more about the genetic architecture of IIM.

## Acknowledgements

None.

### Financial support and sponsorship

S.R., H.C. and J.A.L. are supported by a grant from the Medical Research Council (MR/N003322/1) and the UK Myositis Support Group. S.R. is supported by a Research Fellowship from The Myositis Association. H.C. was supported by a grant from the NIHR Biomedical Research Centre Funding Scheme. The views expressed in this publication are those of the authors and not necessarily those of the NHS, the National Institute for Health Research or the Department of Health.

### Conflicts of interest

There are no conflicts of interest.

## REFERENCES AND RECOMMENDED READING

Papers of particular interest, published within the annual period of review, have been highlighted as:

▪ of special interest
▪▪ of outstanding interest
